# 

**Published:** 1952-10

**Authors:** 


### Contents

| | | Page |
|---|---|---|
| LIFE FOR THE PREMATURE BABY . . . | *Dr. Beryl Corner* | 117 |
| RECENT TRENDS IN BLOOD TRANSFUSION AND RESUSCITATION . . . . . . . . | *Dr. Geoffrey Tovey* | 125 |
| TUBERCULOUS MENINGITIS . . . | *Dr. G. D. W. McKendrick* | 130 |
| THE RESULTS IN 178 CASES OF PREFRONTAL LEUCOTOMY AT THE BRISTOL MENTAL HOSPITALS . | *Dr. Edward H. Hare* | 134 |
| THE WILLIAM BUDD HEALTH CENTRE . . . . . | | 141 |
| ARGYRIA FROM THE USE OF NASAL DROPS . . . . . | | 143 |
| REPORTS OF MEETINGS . . . . . . . . . . . . | | 145 |
| BRISTOL MEDICO-CHIRURGICAL SOCIETY | | |
| DEVON AND EXETER MEDICO-CHIRURGICAL SOCIETY | | |
| SOUTH WEST ORTHOPAEDIC CLUB | | |
| SOUTH WEST PAEDIATRIC CLUB | | |
| APPOINTMENTS . . . . . . . . . . . . . | | 148 |
| NOTES AND NEWS . . . . . . . . . . . . | | 151 |
| EXAMINATION SUCCESSES | | |
| THE TEACHING OF ANAESTHESIA IN EUROPE | | |
| DEGREES OF M.B., CH.B., BRISTOL | | |
| UNIVERSITY OF BRISTOL MEDICAL POSTGRADUATE DEPARTMENT | | |

